# Acupuncture for diabetic peripheral neuropathy: A systematic review and Bayesian network meta-analysis

**DOI:** 10.1097/MD.0000000000043796

**Published:** 2025-08-08

**Authors:** Shuxiong Lin, Yu Qin, Miaomiao Li, Maohuai Zhu, Hao Wen, Yifen Liu, Huijing Lin, Liming Lu

**Affiliations:** a Medical College of Acu-Moxi and Rehabilitation, Guangzhou University of Chinese Medicine, Guangzhou, Guangdong Province, China; b Huizhou Hospital of Traditional Chinese Medicine, Huizhou, Guangdong Province, China; c The Affiliated Huizhou Hospital, Guangzhou Medical University, Huizhou, Guangdong Province, China; d South China Research Center for Acupuncture and Moxibustion, Guangzhou University of Chinese Medicine, Guangzhou, Guangdong Province, China.

**Keywords:** acupuncture, diabetic peripheral neuropathy, multiple interventions, network meta-analysis, type 2 diabetes mellitus

## Abstract

**Background:**

Diabetic peripheral neuropathy (DPN) has emerged as a global health challenge with increasing prevalence rates over the past 3 decades. Acupuncture has been increasingly utilized for the treatment of DPN in recent years. However, whether any specific acupuncture intervention should be considered a priority in the treatment of patients is still unclear.We aimed to summarize the latest evidence concerning the benefits and harms of acupuncture-related therapies to identify an optimal acupuncture intervention for DPN patients.

**Methods:**

This systematic review and network meta-analysis searched databases from inception to October 2024 for randomized controlled trials (RCTs) evaluating acupuncture interventions in patients with DPN receiving mecobalamin therapy. We performed random-effects Bayesian network meta-analyses to synthesize evidence from eligible RCTs.

**Results:**

Our systematic search identified 1831 citations with 62 eligible RCTs involving 5942 participants. Electroacupuncture may be the most effective at improving motor nerve conduction velocity (mean difference [MD]: 10.65; 95% confidence interval [CI]: 4.6–16.7), followed by acupoint injection (AI) combined with traditional Chinese medicine (TCM), AI, herbal fumigation (HF), manual acupuncture (MA), MA combined with HF, and MA combined with moxibustion, with MDs of (10.37; 95% CI: 6.17–14.61), (4.67; 95% CI: 2.57–6.82), (4.72; 95% CI: 1.31–8.14), (3.36; 95% CI: 2.1–4.64), (5.93; 95% CI: 2.53–9.33), and (4.8; 95% CI: 1.69–7.94). AI may be the most effective at improving sensory nerve conduction velocity (MD: 3.94; 95% CI: 2.33–5.57), followed by acupoint injection combined with TCM, bloom needle, HF, MA, MA combined with HF, MA combined with moxibustion, and MA combined with TCM, with MDs of 8.69 (95% CI: 3.72–13.61), 5.6 (95% CI: 2.2–8.99), 4.58 (95% CI: 1.49–7.71), 3.72 (95% CI: 2.62–4.85), 25 (95% CI: 1.64–6.85), 3.58 (95% CI: 1.13–6.08), and 5.7 (95% CI: 4.24–7.17), with low certainty evidence.

**Conclusion:**

Electroacupuncture may be the most effective therapy for improving motor nerve conduction function, and AI may be the best therapy for improving sensory nerve conduction function in patients with DPN.

## 1. Introduction

Diabetic peripheral neuropathy (DPN) has emerged as a global health challenge with increasing prevalence rates over the past 3 decades. Epidemiological projections indicate that approximately 629 million individuals worldwide will be affected by diabetes mellitus by 2045, with 10% to 20% developing DPN as a debilitating complication.^[1]^ This condition manifests as progressive peripheral nerve damage, characterized by distal symmetrical numbness, neuropathic pain, hyperalgesia, and motor dysfunction, predominantly in the lower extremities.^[2]^ The clinical sequelae extend beyond sensory–motor impairments, including diminished walking endurance, sleep disturbances, and emotional dysregulation, collectively contributing to substantial disability-adjusted life years lost.^[3–7]^ Current therapeutic paradigms emphasize pharmacological management, with the recommendation of alpha-lipoic acid, anticonvulsants, antidepressants, and mecobalamine as the first-line agents. In particular, mecobalamines are widely used.^[8]^ Although pharmacotherapy has been shown to be effective in reducing DPN symptoms, such as distal symmetrical numbness, pain, hyperalgesia, and muscle weakness, systematic reviews have revealed that 38% to 45% of patients exhibit a suboptimal response to pharmacotherapy alone, with residual disability rates exceeding 60% at the 5-year follow-up.^[9–11]^ This therapeutic gap has inspired interest in complementary interventions, particularly acupuncture modalities rooted in the traditional Chinese medicine (TCM) theory.

Acupuncture-related therapies for DPN include manual acupuncture (MA), electroacupuncture (EA), warm acupuncture (WA), and hybrid approaches that combine acupoint stimulation with herbal fumigation (HF) or moxibustion (MOX).^[8]^ Mechanistic studies have suggested that these interventions may modulate neuroinflammation via TNF-α/NF-κB pathway inhibition and enhance nerve conduction velocity through BDNF upregulation.^[12]^ However, whether any specific acupuncture intervention should be considered a priority in the treatment of patients is still unclear. Conventional pairwise meta-analyses face inherent limitations in evaluating complex intervention networks, whereas heterogeneity in treatment protocols and outcome measures obscures their comparative effectiveness.

In this study, we conducted a systematic review and network meta-analysis, also known as a multiple-treatment meta-analysis, to compare and rank the efficacy of acupuncture interventions in treating patients with DPN with that of standard regimens by integrating data from direct and indirect evidence.

## 2. Material and methods

This network meta-analysis was registered in the International Prospective Register of Systematic Reviews (PROSPERO; CRD42024583029) and was reported according to the Preferred Reporting Items for Systematic Reviews and Meta-Analyses guidelines for network meta-analysis extension statements (see Supporting Information S1 for the PRISMA checklist, Supplemental Digital Content, https://links.lww.com/MD/P617).

### 2.1. Search strategy

To identify relevant randomized controlled trials (RCTs), we searched the Cochrane Central Register of Controlled Trials, Medline, Embase, and Web of Science from the date of database inception to 2024. We also manually searched for relevant RCTs from the reference lists of the retrieved articles. To supplement the database search, we searched for 2 trial registries (ClinicalTrials.gov and WHO International Clinical Trials Registry Platform). All searches had no restrictions on the date, language, or publication status.

### 2.2. Eligibility criteria

#### 2.2.1. Participants

The target population consisted of patients with clinically confirmed DPN who had received standardized therapy. The enrolled participants were diagnosed with DPN according to the Guidelines for the Prevention and Treatment of Type 2 Diabetes in China (2020 edition) formulated by the Chinese Diabetes Society or the 10th revision of the International Classification of Diseases criteria (ICD-10). Based on the diagnostic criteria for DPN, participants were recruited when they met the following criteria.

(1) Confirmed diagnosis of type 2 diabetes mellitus.
(2) Presence of typical neuropathic symptoms (e.g., distal limb numbness, tingling, burning pain, or decreased sensation), other objective neuropathic symptoms (e.g., reduced conduction velocity or amplitude), or clinical signs (e.g., decreased ankle reflexes, impaired vibration perception).
(3) Exclusion of other causes of neuropathy (e.g., alcohol abuse, vitamin B12 deficiency, autoimmune disorders).

Pregnant women, individuals with severe systemic diseases (e.g., advanced renal failure, malignancy), active infections affecting neurological assessments, and participants with nondiabetic neuropathies or cognitive impairment were excluded.

#### 2.2.2. Intervention

In the standard care protocol for DPN, all participants received conventional therapy, including glycemic control (e.g., insulin or oral hypoglycemic agents) and neurotrophic medications (e.g., mecobalamine, alpha-lipoic acid, or methylcobalamin), to stabilize blood glucose levels and alleviate neuropathic symptoms. Considering the foundational role of glycemic management in DPN progression, patients in both the experimental and control groups received standard care throughout the study. MA, EA, warm acupuncture, acupoint injection (AI) (e.g., the use of vitamin B12), auricular acupuncture, and other acupuncture-related therapies were permitted as experimental therapies (Table 1 and Supporting Information S1, Supplemental Digital Content, https://links.lww.com/MD/P617). The control group received standard care alone, regardless of whether they received additional acupuncture therapies.

**Table 1:** Characteristics of the included randomized controlled trials.

Num.	First author (year)	Diagnostic criteria	Treatments	No. of patients	Treatment duration	Age, mean (SD)	Proportion male/female (%)	Area recruited from	Setting
1	Ye^[13]^	PrMAtical internal medicine (M) 2013	MA-moxibustion versus mecobalamine	47:46	4 weeks	72.13 (4.25); 72.9 (3.73)	59.6; 56.5	Shanxi, China	Inpatient
2	Chen et al^[14]^	Diagnostic criteria for type 2 diabetes (WHO1999)	Moxibustion versus mecobalamine	24:24	9 weeks	61; 60	50; 45.8	Guangdong, China	Inpatient
3	Xiaofeng^[15]^	Diagnostic criteria for type 2 diabetes (WHO1999)	Moxibustion versus mecobalamine	40:40	12 weeks	59. 47 (9.32); 60. 88 (8.49)	57.5; 55	Henan, China	Outpatient
4	Yao et al^[16]^	Diagnostic criteria for diabetes (ADA,1997)	MA versus mecobalamine	40:40	4–8 weeks	54.5; 53.4	60; 52.5	Jiangxi, China	Outpatient and inpatient
5	Li et al^[17]^	Clinical diabetology (1989)	BL versus mecobalamine	32:26	8 weeks	58.32 (8.23); 57.68 (8.18)	59.4; 57.7	Anhui, China	Outpatient and inpatient
6	Liu et al^[18]^	Chinese guidelines for the prevention and treatment of type 2 diabetes (2013 edition)	AI versus mecobalamine	50:50	8 weeks	57.44 (12.30); 58.03 (12.78)	48; 50	Henan, China	Outpatient and inpatient
7	Su^[19]^	Diagnostic criteria for type 2 diabetes (WHO1999)	AI versus mecobalamine	60:60	2 weeks	52.29 (5.45); 53.12 (4.99)	46.7; 51.7	Guangxi, China	Inpatient
8	Wu et al^[20]^	Diagnostic criteria for type 2 diabetes (WHO1999)	Moxibustion versus mecobalamine	26:26	4 weeks	60.69 (7.84); 58.76 (7.92)	53.8; 50	Hebei, China	Inpatient
9	Cui^[21]^	Diagnostic criteria for type 2 diabetes (WHO1999)	EA versus MA	30:30		57.85 (5.40); 57.93 (5.46)	70; 63.3	Jiangsu, China	Inpatient
10	Ren et al^[22]^	Guidelines for diagnosis and treatment of diabetic peripheral neuropathy (draft for Comment2009)	MA versus mecobalamine	30:30	8 weeks	53.89 (7.74); 55.26 (6.91)	46.7; 50	Heilongjiang, China	Inpatient
11	Zhou and Yang^[23]^	Diagnostic criteria for type 2 diabetes (WHO1999)	MA-TCM versus MA	48:48	8 weeks	51.1 (6.1); 50.3 (5.9)	54.2; 56.3	Shanxi, China	Inpatient
12	Jingsong^[24]^	Chinese guidelines for the prevention and treatment of type 2 diabetes (2013 edition)	MA-TCM versus mecobalamine	48:47	8 weeks	65.4 (5.1); 63.6 (6.2)	56.3; 59.6	Beijing, China	Outpatient
13	Song et al^[25]^	Chinese guidelines for the prevention and treatment of type 2 diabetes (2013 edition)	MA+HF versus mecobalamine	30:30	2 weeks	56.40 (6.55); 57.77 (7.20)	46.7; 40	Ningxia, China	Inpatient
14	Tao et al^[26]^	Guidelines for the prevention and treatment of diabetic peripheral neuropathy (2011)	MA-TCM versus mecobalamine	70:70	6 weeks	55.3 (12.7); 56.2 (11.9)	64.3; 61.4	Hebei, China	Inpatient
15	Mo et al^[27]^	Diagnostic criteria for type 2 diabetes (WHO1999)	AI versus mecobalamine	40:42	2 weeks	58.85 (7.60); 59.17 (8.01)	55; 52.4	Guangxi, China	Inpatient
16	Wei et al^[28]^	Chinese type 2 diabetes prevention and control Index South (2017 edition)	AI-TCM versus AI	100:100	4 weeks	57.25 (4.55); 57.37 (4.25)	57; 58	Henan, China	Inpatient
17	Li et al^[29]^	Diagnostic criteria for type 2 diabetes (WHO2009)	MA versus mecobalamine	20:20	4 weeks	Not mention	45	Heilongjiang, China	Outpatient
18	Zheng et al^[30]^	Guidelines for diagnosis and treatment of diabetic peripheral neuropathy (draft for Comment2009)	Moxibustion-TCM versus TCM	36:33	12 weeks	59.1 (4.3); 58.4(4.6)	55.5; 54.5	Beijing, China	Outpatient
19	Zhou et al^[31]^	Diagnosis and treatment of diabetic peripheral neuropathy (draft for Comment2009)	MA versus AI versus mecobalamine	104:104:104	3 weeks	57.18 (6.69); 56.98(6.47); 57.56 (6.62)	57.7; 65.4; 67.3	Hebei, China	Inpatient
20	Jing^[32]^	–	BN versus mecobalamine	60:50	4 weeks	62.73 (6.21); 62.93 (4.19)	56.7; 56	Guangdong, China	Inpatient
21	Chen and Yu^[33]^	Chinese guidelines for the prevention and treatment of type 2 diabetes (2013 edition)	AI versus mecobalamine	47:49	4 weeks	52.32 (10.51); 53.09 (12.52)	46.8; 54.2	Hubei, China	Inpatient
22	Yi et al^[34]^	Chinese guidelines for the prevention and treatment of type 2 diabetes (2017 edition)	MA-TCM versus MA versus TCM	33:33:33	4 weeks	55.88 (7.98); 52.79 (8.44); 53.45 (9.44)	51.5; 57.8; 54.5	Anhui, China	Inpatient
23	Zhao^[35]^	Chinese guidelines for the prevention and treatment of type 2 diabetes (2020 edition)	MA-moxibustion versus mecobalamine	40:40	12 weeks	66.75 (6.41); 65.48 (8.09)	60; 57.4	Guangxi, China	Inpatient
24	Zhou et al^[36]^	Chinese guidelines for the prevention and treatment of type 2 diabetes (2013 edition)	BL versus mecobalamine	30:30	4 weeks	54.32 (8.23); 56.75 (7.46)	53.3; 50	Guangdong, China	Outpatient
25	Jin et al^[37]^	Diabetic peripheral neuropathy TCM clinical diagnosis and treatment guidelines (2016 edition)	WA versus mecobalamine	30:30	12 weeks	62.18 (6.10); 61.87 (5.92)	60; 66.6	Shanghai, China	Outpatient
26	Liu et al^[38]^	2010 ADA diabetes guidelines	MA versus AI versus PA	15:15:15	2 weeks	Not mention	46.7; 60; 60	Guangdong, China	Outpatient and inpatient
27	Jing et al^[39]^	China type 2 diabetes prevention and treatment guidelines (2020 edition)	MA+HF versus HF versus MA versus mecobalamine	34:34:33:33	8 weeks	55.91 (7.03); 55.29 (6.81); 53.85 (6.34); 54.68 (5.72)	58.8; 55.9; 57.6; 54.5	Henan, China	Inpatient
28	Du et al^[40]^	Clinical guidelines for diabetes Mellitus(2000)	WA versus mecobalamine	40:40	5 weeks	54.74 (8.19); 54.67 (7.03)	67.5; 57.5	Neimenggu, China	Inpatient
29	Ma et al^[41]^	First draft of TCM diagnosis and treatment standards for diabetic peripheral neuropathy (2010)	WA versus MA	30:30	4 weeks	55 (6); 56 (5)	50; 46.9	Heilongjiang, China	Outpatient
30	Sun and Xu^[42]^	Diagnostic criteria for type 2 diabetes (WHO1999)	WA versus mecobalamine	26:26	4 weeks	61.00 (9.27); 60.70 (6.56)	57.7; 65.4	Heilongjiang, China	Outpatient and inpatient
31	Xin et al^[43]^	Diagnostic criteria for type 2 diabetes (WHO1999)	AI-TCM versus AI	43:43	4 weeks	54.81 (8.57)	44.2; 51.1	Guangdong, China	Outpatient
32	Yuai et al^[44]^	Chinese guidelines for the prevention and treatment of type 2 diabetes (2013 edition)	AI versus mecobalamine	60:60	4 weeks	59 (3); 59 (3)	55; 53.3	Hebei, China	Inpatient
33	Wang et al^[45]^	PrMAtical internal medicine	Moxibustion versus mecobalamine	30:30	4 weeks	Not mention	46.6	Anhui, China	Inpatient
34	Yanqiu and Wenwang^[46]^	Expert consensus on diagnosis and treatment of diabetic neuropathy (2021 edition)	MA-TCM versus MA	48:48	2 weeks	57.26 (9.83); 55.94 (9.32)	52.1; 58.3	Anhui, China	Inpatient
35	Huijing et al^[47]^	Consensus on diagnosis and treatment of diabetic peripheral neuropathy [J].2013	MA-TCM versus MA versus TCM	53:54:53	8 weeks	54.23 (13.52); 53.85 (12.14); 54.43 (13.97)	54.7; 53.7; 56.6	Guangdong, China	Inpatient
36	Zhao and Zhang^[48]^	Diagnostic criteria for diabetes (ANA1997)	MA versus mecobalamine	30:30	8 weeks	53 (9.2)	58.3	Shanxi, China	Inpatient
37	Qiqi et al^[49]^	Diabetic neuropathy: a position statement by the American Diabetes Association (2017)	MA versus mecobalamine	25:25	4 weeks	57.24 (7.18); 53.92 (5.97)	40; 48	Jiangsu, China	Inpatient
38	Li and Yang^[50]^	Diagnostic criteria for type 2 diabetes (WHO1999)	MA-TCM versus MA	55:50	8 weeks	58; 56	63.6; 60	Jilin, China	Outpatient and inpatient
39	Zhang et al^[51]^	Diagnostic criteria for diabetes (ANA1997)	MA-HF versus mecobalamine	36:36	4 weeks	Not mention	52.7; 50	Heilongjiang, China	Outpatient and inpatient
40	Cao et al^[52]^	Chinese guidelines for the prevention and treatment of type 2 diabetes (2017 edition)	MA-TCM versus MA	78:78	4 weeks	65.07 (10.35); 64.60 (9.90)	55.1; 51.3	Hebei, China	Outpatient and inpatient
41	Jihong^[53]^	Diagnostic criteria for type 2 diabetes (WHO1999)	MA versus mecobalamine	46:42	8 weeks	Not mention	52.2; 52.4	Henan, China	Inpatient
42	Ren et al^[54]^	Diagnostic criteria for type 2 diabetes (WHO1999)	MA-TCM versus MA	25:25	4 weeks	Not mention	Not mention	Heilongjiang, China	Inpatient
43	Chen et al^[55]^	Diagnostic criteria for type 2 diabetes (WHO1999)	EA versus mecobalamine	34:29	2 weeks	68.55 (8.91)	60.6	Shanghai, China	Inpatient
44	Wang et al^[56]^	Diagnostic criteria for type 2 diabetes (WHO1999)	MA versus mecobalamine	34:32	4 weeks	56.10 (5.33); 58.45 (8.52)	58.8; 56.3	Heilongjiang, China	Outpatient and inpatient
45	Yadong et al^[57]^	Diabetic peripheral neuropathy TCM clinical diagnosis and treatment guidelines (2016 edition)	MA versus mecobalamine	75:75	4 weeks	60.23 (7.54); 59.48 (7.63)	53.3; 54.6	Shanxi, China	Inpatient
46	He et al^[58]^	Diagnostic criteria for type 2 diabetes (WHO1999)	EA versus mecobalamine	42:36	4 weeks	55.3 (2.6); 53.9 (1.9)	47.6; 44.4	Guangdong, China	Outpatient and inpatient
47	Li et al^[59]^	Chinese guidelines for the prevention and treatment of type 2 diabetes (2017 edition)	MA versus mecobalamine	45:45	3 weeks	58 (9); 59 (7)	64.4; 60	Hebei, China	Inpatient
48	Wang et al^[60]^	Internal medicine [M].2013	MA-moxibustion versus mecobalamine	79:78	8 weeks	63.5 (6.9); 64.7 (7.2)	53.2; 51.3	Beijing, China	Inpatient
49	Li et al^[61]^	Diagnostic criteria for type 2 diabetes (WHO1999)	MA versus mecobalamine	56:56	3 weeks	55 (10); 55 (8)	51.8; 55.4	Hebei, China	Inpatient
50	Zheng et al^[62]^	Chinese guidelines for the prevention and treatment of type 2 diabetes (2007)	MA versus mecobalamine	31:30	2 weeks	56.42 (10.01); 55.38 (9.65)	56.3; 50	Heilongjiang, China	Inpatient
51	Liu^[63]^	Diagnostic criteria for type 2 diabetes (WHO1999)	MA-moxibustion versus mecobalamine	32:32	12 weeks	54.32 (9.72); 63.78 (10.13)	43.8; 53.1	Guangdong, China	Outpatient and inpatient
52	Li and Yang^[50]^	New diagnostic criteria and classification of diabetes mellitus	MA versus mecobalamine	30:30	16 weeks	56.1 (3.2)	61.7	Shandong, China	Outpatient and inpatient
53	Guangsheng^[64]^	Diagnostic criteria for type 2 diabetes (WHO1999)	MA-TCM versus mecobalamine	44:42	8 weeks	58.4 (5.8); 58.1 (6.0)	54.5; 54.8	Henan, China	Not mention
54	Hou et al^[65]^	Chinese guidelines for the prevention and treatment of type 2 diabetes (2013 edition)	MA-TCM versus mecobalamine	40:40	4 weeks	54.92 (10.65); 59.76 (9.43)	47.5; 50	Guizhou, China	Outpatient and inpatient
55	Liu^[66]^	Diagnostic criteria for type 2 diabetes (WHO1999)	MA-TCM versus TCM	160:120	8 weeks	Not mention	52.5	Henan, China	Not mention
56	Gao et al^[67]^	Diagnostic criteria for type 2 diabetes (WHO1999)	MA-TCM versus mecobalamine	80:80	4 weeks	58.4 (4.8)	53.8	Shamxi, China	Inpatient
57	Hu and Luo^[68]^	Diabetes screening and diagnosis [S]. 2012	MA-TCM versus mecobalamine	49:40	12 weeks	57.3 (4.6)	47.2	Zhejiang, China	Inpatient
58	Chen^[69]^	Diagnostic criteria for type 2 diabetes (WHO1999)	AI versus mecobalamine	87:87	6 weeks	51.9 (7.4); 52.3 (7.9)	52.9; 51.7	Hainan, China	Inpatient
59	Yao^[70]^	Chinese guidelines for the prevention and treatment of type 2 diabetes (2013 edition)	AI versus mecobalamine	38:38	4 weeks	57.19 (6.08); 56.28 (5.63)	44.7; 47.3	Liaoning, China	Inpatient
60	Xu et al^[71]^	Chinese guidelines for the prevention and treatment of type 2 diabetes (2010 edition)	HF versus mecobalamine	34:34	2 weeks	46.98 (6.37); 48.25 (7.64)	61.8; 67.6	Jiangsu, China	Inpatient
61	Liu^[18]^	Chinese guidelines for the prevention and treatment of type 2 diabetes (2010 edition)	BN versus mecobalamine	53:53	8 weeks	61.13 (4.66); 59.91 (4.47)	58.5; 54.7	Anhui, China	Inpatient and out patient
62	Wang and Guo^[72]^	Not mention	MA versus mecobalamine	50:46	8 weeks	60.28 (8.74); 61.64 (7.33)	56; 36.96	Tianjin, China	Not mention

AI = acupoint injection, AI-TCM = acupoint injection combined with traditional Chinese medicine, BL = blood-letting, BN = bloom needle, EA = electroacupuncture, HF = herbal fumigation, MA = manual acupuncture, MA-HF = manual acupuncture combined with herbal fumigation, MA-MOX = manual acupuncture combined with moxibustion, MA-TCM = manual acupuncture combined with traditional Chinese medicine, MEC = mecobalamin, MOX = moxibustion, MOX-TCM = moxibustion combined with traditional Chinese medicine, PA = placebo acupuncture, TCM = traditional Chinese medicine, WA = warming acupuncture.

#### 2.2.3. Outcomes

We considered motor nerve conduction velocity (MNCV) as the primary outcome in the trials, as it objectively reflects the functional integrity of large myelinated fibers and correlates with the clinical progression of motor impairment. Given the chronic nature of DPN, we prioritized long-term electrophysiological outcomes to capture sustained treatment effects. Therefore, the primary outcome was the change in the MNCV (expressed in meters per second, m/s) of the peroneal nerve, which was measured via standardized nerve conduction studies using surface electrodes.

The secondary outcome was sensory nerve conduction velocity (SNCV) of the sural nerve (m/s), which complements motor assessments by evaluating small-fiber dysfunction.

### 2.3. Selection of studies and data extraction

EndNote and Microsoft Excel were used to manage all search results. Four authors (S.X.L., Y.Q., M.H.Z., and H.W.) independently screened the titles and abstracts of the identified references. All potentially eligible references were obtained as full text and screened independently by S.X.L., Y.Q., and M.H.Z. A predefined data extraction sheet was used to extract data from eligible references. The data extraction researchers discussed the conflicts of opinion, which were resolved by another member of the study team.

Two independent reviewers (S.X.L. and M.M.L.) conducted the data extraction using a standardized protocol to ensure methodological rigor. We extracted data on study characteristics (author, design, population demographics), intervention details (acupuncture type, parameters [e.g., frequency, duration], acupoint selection), outcomes (motor and sensory nerve conduction velocities with measurement protocols), cross-validated results via line-line comparisons, and resolved discrepancies through consensus or third-party arbitration. Missing data were sought through author contact, with unresolved items marked “unclear.”

### 2.4. Risk of bias assessment

Methodological quality was systematically appraised using the Cochrane Risk of Bias Tool (RoB 1.0), with independent assessments conducted across 6 critical domains: (1) randomization sequence generation, (2) allocation concealment, (3) blinding of participants, (4) blinding of outcome assessment, (5) completeness of outcome data, (6) selective reporting bias, and (7) other bias. Inter-rater discrepancies in quality appraisal or data interpretation were resolved through iterative consensus discussions, with persistent disagreements adjudicated by a senior methodologist (M.H.Z.). This rigorous implementation of the Cochrane framework ensured a standardized evaluation of study validity while maintaining procedural transparency throughout the evidence synthesis process.

### 2.5. Assessment of the certainty of evidence

The GRADE method was used to assess the overall quality of the evidence for each outcome. The quality of evidence was downgraded from high quality by one level for serious issues (or 2 levels for very serious issues) related to study limitations, inconsistency of effect, indirectness of evidence, imprecision of effect estimates, or publication bias. A higher level of certainty of evidence indicated a lower likelihood of changing the results based on future investigations. Conversely, a lower level of certainty of evidence suggested a greater susceptibility to result changes.The evidence body was assessed by 2 individuals (M.M.L. and S.X.L.), and their assessments were cross-validated. Disagreements were resolved through discussion.

### 2.6. Statistical analysis

A classic meta-analysis was performed to evaluate the efficacy of acupuncture-related therapies for improving neural function in patients with DPN. To assess the statistical heterogeneity in each pairwise comparison, we calculated the *I*^2^ statistic using the Cochran Q test. *P* > .1 or *I*^2^ < 50% was considered to indicate heterogeneity, and a random-effects model was used. Otherwise, a fixed-effects model was used. A sensitivity analysis was used to clarify the source of heterogeneity. Funnel plots were used to detect publication biases.

We incorporated indirect comparisons with direct comparisons via random-effects network meta-analyses within a Bayesian network framework with a Monte Carlo Markov chain model via “gemtc 0.8--7” and its dependent packages in R software (version 4.0.5, the R Foundation, https://www.r-project.org). We simultaneously conducted 4 Monte Carlo Markov chain models, and the number of simulations was set to 20,000, with the number of iterations set to 100,000. We constructed fixed-effects models and random-effects models to evaluate the fitting effect of different models on the data based on the deviation information criterion and diagnostic parameters potential scale reduction factor (PSRF) from Brooks-Gelman-Rubin. If the PSRF value is close to one and the deviation information criterion value is lower, the model convergence is more satisfactory.

The network geometry was constructed via a graph-theoretical approach, where nodes represented distinct therapeutic interventions (e.g., EA, MA, and standard pharmacotherapy) and edges denoted direct head-to-head comparisons identified from the included RCTs. All results of the network meta-analyses for binary outcomes are presented in league tables with effect sizes (mean difference [MD]), *P* values (with a family wise alpha level of .05), and 95% confidence intervals (CIs) (according to whether the CI included the null value) to assess significance. We also assessed the ranking probabilities for all acupuncture interventions by calculating the MD for each acupuncture intervention compared with any control group and counting the proportion of iterations of the Markov chain in which each acupuncture intervention had the highest MD, the second highest, the third highest, etc.

The coherent assumption behind network meta-analysis is a key assumption: that direct and indirect evidence on the same comparisons do not disagree beyond chance; thus, we assessed incoherence between direct and indirect sources of evidence via node-splitting analysis.

## 3. Results

### 3.1. Study identification and selection

**Figure 1.**
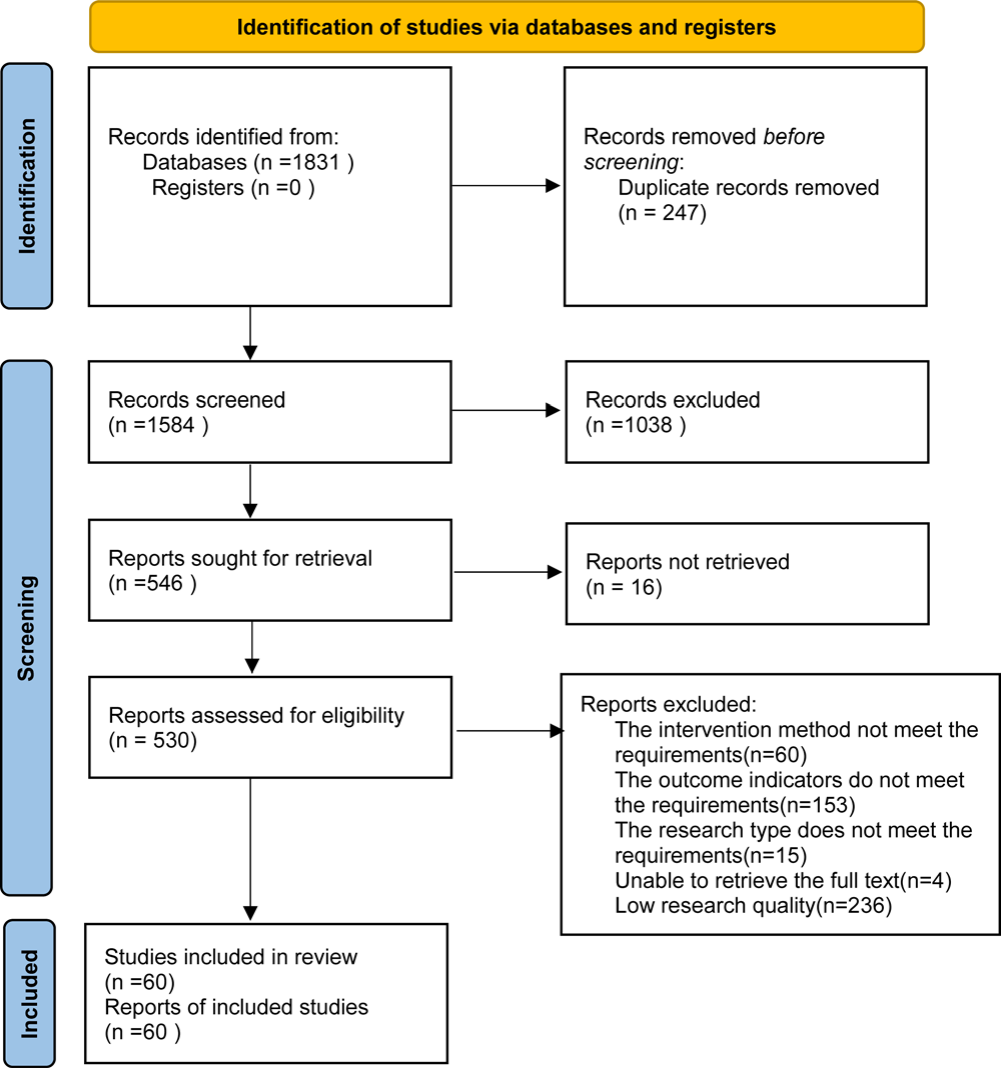
Flow diagram.

Overall, 1831 studies were identified from the 7 electronic databases using the search strategy, and 530 relevant full-text articles were evaluated for eligibility. A total of 468 citations were excluded, including 60 for the intervention method that did not meet the requirements, 153 for the outcomes that did not meet the requirements, 15 for the research type that did not meet the requirements, 4 for inability to retrieve the full test, and 236 for low research quality. Ultimately, 62 studies^[13–74]^ including 5942 patients were clinically eligible for inclusion in this network meta-analysis (Fig. 1). The characteristics of the selected studies are presented in Table 1.

### 3.2. Risk of bias assessment

**Figure 2.**
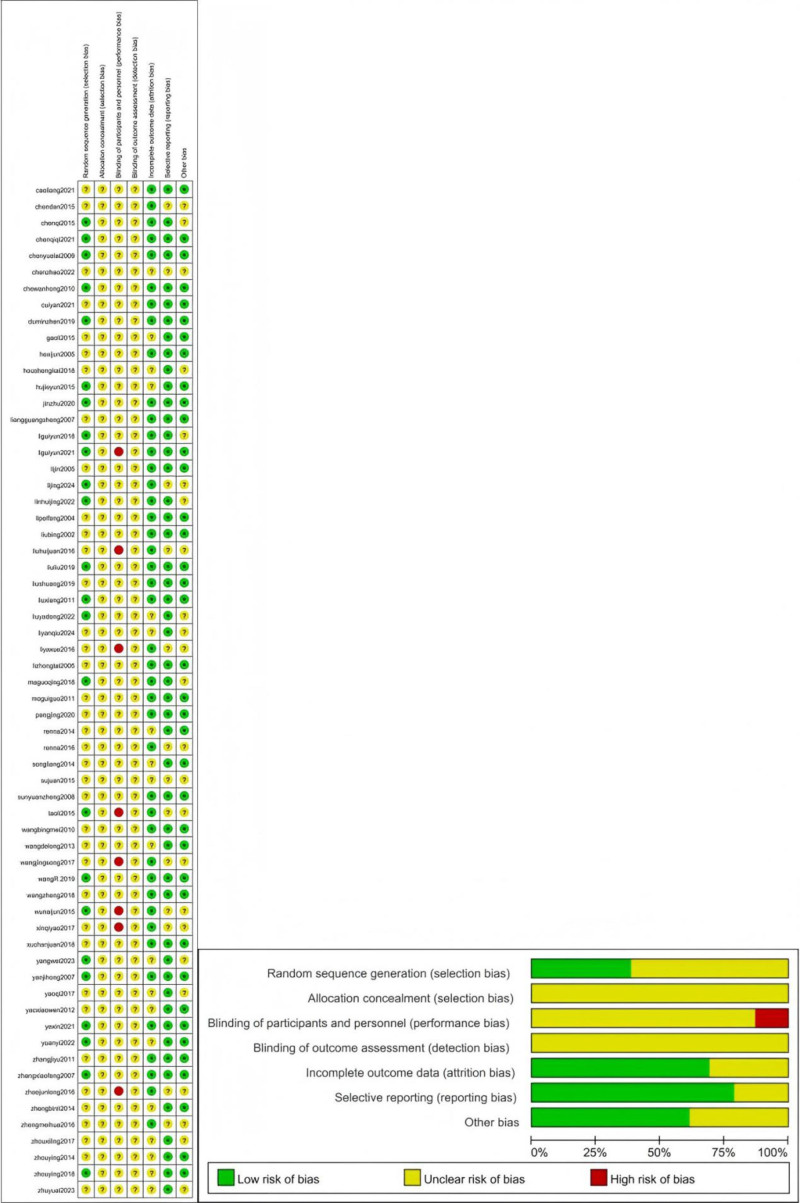
Risk of bias.

The high overall risk of bias (812.9%) occurred predominantly in the domains of performance bias because it was difficult to blind the participants to group allocation. The low or unclear overall risk of bias (5487.1%) occurred because of insufficient reporting of randomization, allocation concealment, or blinding of the outcome assessment. The reviewers showed substantial agreement in 7 domains (Fig. 2).

### 3.3. Certainty of evidence

The GRADE approach was used to assess the certainty of evidence for both primary and secondary outcomes. The results revealed low-quality evidence for the primary and secondary outcomes, indicating that acupuncture plus conventional therapy may increase or have little to no effect on MNCV/SNCV. However, evidence for this is uncertain. For details on the ratings, see Table 2.

**Table 2:** GRADE evidence profile of acupuncture combined with conventional therapy for MNCV and SNCV.

Certainty assessment							No of patients		Effect	Certainty	Importance
No of studies	Study design	Risk of bias	Inconsistency	Indirectness	Imprecision	Other considerations	Manual acupuncture	Sham acupuncture	MD (95% CI)		
**(A) MNCV (follow-up: range 2 weeks to 16 weeks; assessed with: standardized nerve conduction studies**)											
56	Randomized trials	Serious*	No serious	Not serious	Serious†	None	2676	2584	3.89 (3.36 higher to 4.43 higher)	Low*^,^†	Critical
(B) **SNCV (follow-up: range 2 weeks to 16 weeks; assessed with: standardized nerve conduction studies**)											
46	Randomized trials	Serious*	No serious	Not serious	Serious†	None	2204	2161	4.05 (3.52 higher to 4.59 higher)	Low*^,^†	Important

GRADE certainty of evidence: low certainty.

CI = confidence interval, MD = mean difference, MNCV = motor nerve conduction velocity, SNCV = sensory nerve conduction velocity.

* Most of the included randomized controlled trials had an unclear risk of concealment of allocation.

† The sample size was <200; the number of events was not high; 95% CIs showed an overlap and crossed the line of no effect and appreciable benefit.

### 3.4. Meta-analysis

**Figure 3.**
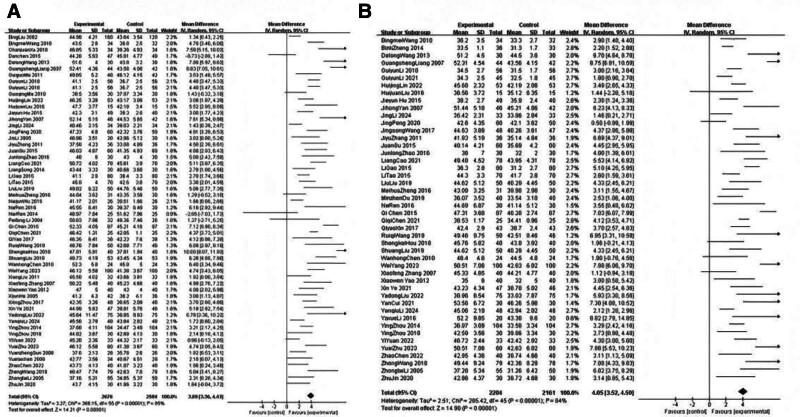
Forest plots of MNCV and SNCV in patients with DPN. (A) Forest plots of MNCV values. (B) Forest plots of the SNCV. DPN = diabetic peripheral neuropathy, MNCV = motor nerve conduction velocity, SNCV = sensory nerve conduction velocity.

**Figure 4.**
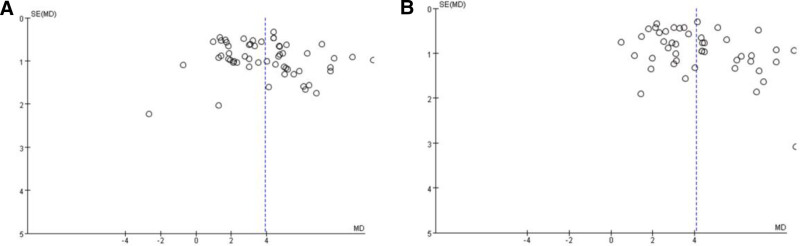
Funnel plots of MNCV and SNCV in patients with DPN. (A) Funnel plots of MNCV values. (B) Funnel plots of SNCV. DPN = diabetic peripheral neuropathy, MNCV = motor nerve conduction velocity, SNCV = sensory nerve conduction velocity.

To measure the efficacy of acupuncture interventions, a classic meta-analysis was performed using a random-effects model to reduce the false-positive rate and control for type I errors. We considered the presence of a statistically significant difference by setting the *P* value to <.05. For the primary outcome, the pooled results of 56 trials revealed that acupuncture-related therapies resulted in faster MNCV after treatment than conventional therapy (MD 3.89, 95% CI 3.36–4.43; *P* < .00001). For the secondary outcome, the pooled results of 46 trials indicated that acupuncture-related therapies resulted in faster SNCV after treatment than conventional therapy (MD 4.05, 95% CI 3.52–4.59; *P* < .00001). There was substantial heterogeneity between the results (*I*^2^ = 85%, *Chi*^2^ test *P* < .00001; *I*^2^ = 84%, *Chi*^2^ test *P* < .00001), which might be explained by the difference in the participants and acupuncture regimens. Forest plots are shown in Fig. 3. The funnel plot was relatively symmetrical overall, which might indicate that there was no detected potential bias (Fig. 4).

### 3.5. Network meta-analysis

**Figure 5.**
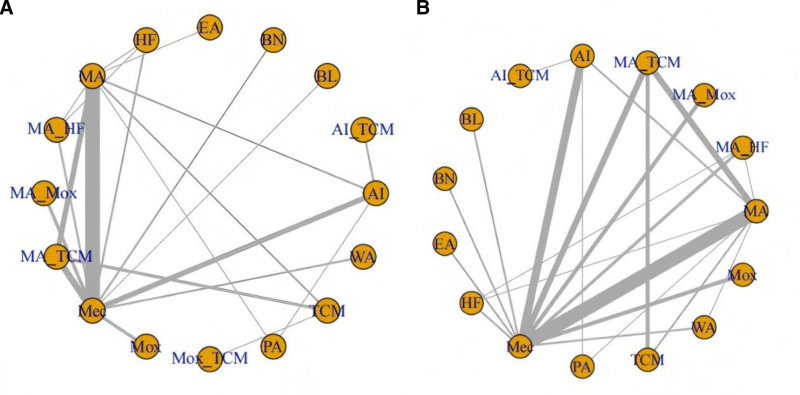
Network of eligible comparisons. (A) Common peroneal nerve sensory nerve; (B) common peroneal nerve motor nerve. AI = acupoint injection, AI-TCM = acupoint injection combined with traditional Chinese medicine, BL = blood letting, BN = bloom needle, EA = electroacupuncture, HF = herbal fumigation, MA = manual acupuncture, MA-HF = manual acupuncture combined with herbal fumigation, MA-MOX = manual acupuncture combined with moxibustion, MA-TCM = manual acupuncture combined with traditional Chinese medicine, MEC = mecobalamin, MOX = moxibustion, MOX-TCM = moxibustion combined with traditional Chinese medicine, PA = placebo acupuncture, TCM = traditional Chinese medicine, WA = warming acupuncture.

We present all the networks for the specific outcomes in Fig. 5. In the network plot, nodes and edges were weighted according to the number of acupuncture interventions and comparisons. The width of the lines was proportional to the number of trials comparing each pair of treatments, and the size of each node was proportional to the number of randomly assigned participants. Sixteen acupuncture interventions had at least one trial versus mecobalamine, and all were directly compared with at least one other acupuncture intervention.

#### 3.5.1. Primary outcome: MNCV

**Figure 6.**
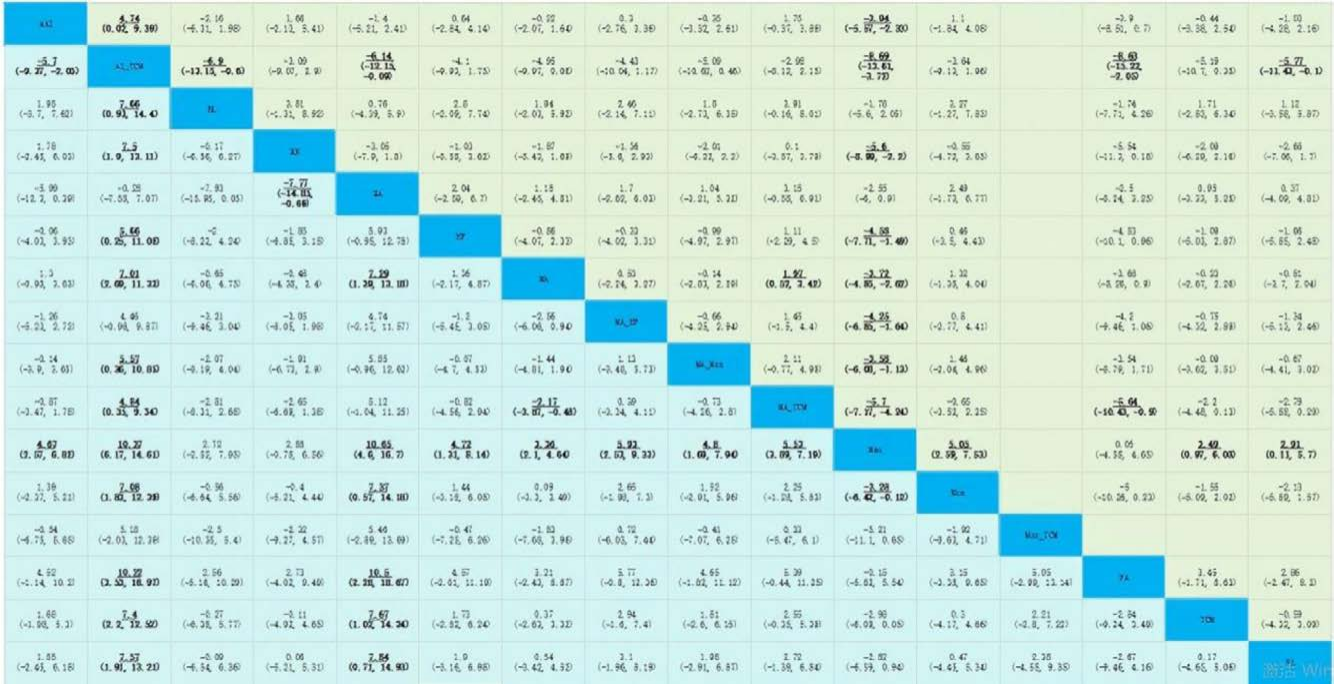
Network meta-analysis of the common peroneal nerve motor nerve (blue) and common peroneal nerve sensory nerve (green). *Notes*: The diagonal shows the different nodes examined in this study. On the left side of the diagonal, the values for the common peroneal nerve motor nerve conduction velocity were given as MDs with a 95% confidence interval (CI). At the right side of the diagonal, the values for the common peroneal nerve sensory nerve conduction velocity are given as the MD with 95% CI; acupuncture interventions are reported in alphabetical order. The results are the MDs in the column-defining treatment compared to the MDs in the row-defining treatment. For the common peroneal nerve motor nerve (blue), MDs >0 favor column-defining acupuncture intervention. For the common peroneal nerve sensory nerve (green), MDs >0 favor row-defining treatment. Reciprocals should be used to obtain the MD for comparison in the opposite direction. The significant results are indicated in bold and underlined. AI = acupoint injection, AI-TCM = acupoint injection combined with traditional Chinese medicine, BL = blood letting, BN = bloom needle, EA = electroacupuncture, HF = herbal fumigation, MA = manual acupuncture, MA-HF = manual acupuncture combined with herbal fumigation, MA-MOX = manual acupuncture combined with moxibustion, MA-TCM = manual acupuncture combined with traditional Chinese medicine, MEC = mecobalamin, MOX = moxibustion, MOX-TCM = moxibustion combined with traditional Chinese medicine, PA = placebo acupuncture, TCM = traditional Chinese medicine, WA = warming acupuncture.

**Figure 7.**
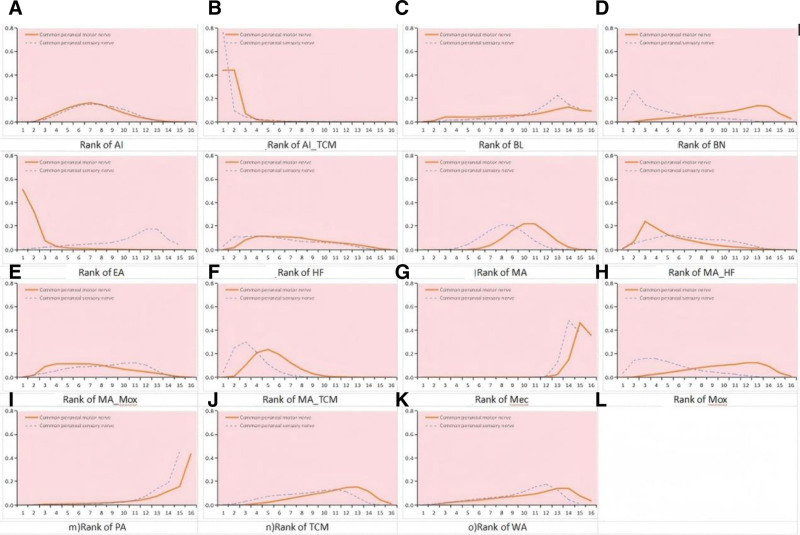
Ranking of the common peroneal nerve sensory nerve (blue dotted line) and common peroneal nerve motor nerve (red solid line). *Notes*: Ranking indicates that probability is the best, second best, third best, and so on, among the 16 acupuncture interventions. AI-TCM = acupoint injection combined with traditional Chinese medicine, BL = blood-letting, BN = bloom needle, EA = electroacupuncture, HF = herbal fumigation, MA = manual acupuncture, MA-HF = manual acupuncture combined with herbal fumigation, MA-MOX = manual acupuncture combined with moxibustion, MA-TCM = manual acupuncture combined with traditional Chinese medicine, MEC = mecobalamin, MOX = moxibustion, MOX-TCM = moxibustion combined with traditional Chinese medicine, PA = placebo acupuncture, TCM = traditional Chinese medicine, WA = warming acupuncture.

We employed node-splitting analysis to evaluate consistency, and all *P*-values comparing the direct and indirect effects were* > *.05. A PSRF value near 1 suggests that the model convergence was more appropriate, indicating stable and reliable results. For the common peroneal nerve motor nerve conduction velocity, the AI (MD: 4.67; 95% CI: 2.57–6.82), AI combined with TCM (AI-TCM) (MD: 10.37; 95% CI: 6.17–14.61), EA (MD:10.65; 95% CI: 4.6–16.7), HF (MD: 4.72; 95% CI: 1.31–8.14), MA (MD: 3.36; 95% CI: 2.1–4.64), MA combined with HF (MA-HF) (MD: 5.93; 95% CI: 2.53–9.33), MA combined with MOX (MA-MOX) (MD: 4.8; 95% CI: 1.69–7.94), MA combined with TCM (MA-TCM) (MD: 5.53; 95% CI: 3.89–7.19), and MOX (MD: 3.28; 95% CI: 0.12–6.42) were statistically more efficient than mecobalamine alone. AI (MD: ‐5.7; 95% CI: ‐9.37 to ‐2.05); blood-letting (MD: ‐7.66; 95% CI: ‐14.4 to ‐0.93); bloom needle (MD: ‐7.5; 95% CI: ‐13.11 to ‐1.9); HF (MD: ‐5.66; 95% CI: ‐11.08 to ‐0.25); MA (MD: ‐7.01; 95% CI: ‐11.33 to ‐2.69); MA-MOX (MD: ‐5.57; 95% CI: ‐10.85 to ‐0.36); MA-TCM (MD: ‐4.84; 95% CI: ‐9.34 to ‐0.35); MOX (MD: ‐7.08; 95% CI: ‐12.39 to ‐1.83); placebo acupuncture (MD: ‐10.22; 95% CI: ‐16.97 to ‐3.53); TCM (MD: ‐7.4; 95% CI: ‐12.52 to ‐2.2); WA (MD: ‐7.57; 95% CI: ‐13.21, see Figs. 5 and 6). In a network meta-analysis, the common peroneal nerve motor nerve outcome ranked EA (51%) as the best outcome, followed by AI-TCM (44%). See Fig. 7.

#### 3.5.2. Secondary outcome: SNCV

In the network meta-analysis, for the common peroneal nerve sensory nerve conduction velocity, the AI (MD: 3.94; 95% CI: 2.33–5.57), AI-TCM (MD: 8.69; 95% CI: 3.72–13.61), bloom needle (MD: 5.6; 95% CI: 2.2–8.99), HF (MD: 4.58; 95% CI: 1.49–7.71), MA (MD: 3.72; 95% CI: 2.62–4.85), MAHF (MD: 4.25; 95% CI: 1.64–6.85), MA-MOX (MD: 3.58; 95% CI: 1.13–6.08), MA-TCM (MD: 5.7; 95% CI: 4.24–7.17), MOX (MD: 5.05; 95% CI: 2.59–7.53), and TCM (MD: 3.49; 95% CI: 0.97–6.08) were statistically more efficient than mecobalamine alone. AI-TCM (MD: 4.74; 95% CI: 0.02–9.39) was significantly more efficient than AI alone. MA-TCM (MD: 1.97; 95% CI: 0.52–3.42) was significantly more efficient than MA alone. Blood-letting (MD: ‐6.9; 95% CI: ‐13.15–0.6), EA (MD: ‐6.14; 95% CI: ‐12.15–0.09), placebo acupuncture (MD: ‐8.63; 95% CI: ‐15.22 to ‐2.05), and WA (MD: ‐5.77; 95% CI: ‐11.43 to ‐0.1) were statistically less efficient than AI-TCM. See Figs. 5 and 6 for details. For the common peroneal sensory nerve outcome, the AI-TCM (76%) was the best. See Fig. 7.

## 4. Discussion

### 4.1. Principal findings

This network meta-analysis of 62 RCTs (n = 5942) demonstrated that AI-TCM and EA exhibited superior efficacy in improving common peroneal nerve function among patients with DPN. Compared to mecobalamine alone, AI, AI-TCM, EA, HF, MA-HF, MA-MOX, MA-TCM, MOX, and TCM were all more effective. The cumulative ranking probabilities indicated that AI-TCM might be the most effective intervention for overall symptom improvement, particularly for sensory function, and that EA showed priority in enhancing motor function. When the sensory function of the common peroneal nerve was compared, EA was not recognized as one of the best treatments in terms of ranking probability, probably because of the following reasons: (a) network meta-analysis revealed no significant difference between acupuncture interventions; (b) low-quality primary studies. However, both direct evidence and network meta-analysis results suggest that EA is more effective than mecobalamine; therefore, we believe that EA might be an effective treatment for improving common peroneal nerve function.

### 4.2. Interpretation of the effects of acupuncture

AI-TCM may effectively alleviate the clinical symptoms of patients, improve microcirculation, and promote nerve tissue.^[70]^ EA may improve nerve conduction speed, reduce the degree of pain, and improve the clinical symptoms of patients, possibly because EA can promote local nerve injury, accelerate the absorption of local degeneration, necrosis, and disintegration products, and help improve clinical symptoms.^[21]^ The effect of AI-TCM may stem from multimodal synergism; AI enhances localized neurotrophic support through drug permeation, whereas systemic regulation via herbal formulations concurrently ameliorates microcirculatory deficits and nerve conduction velocity.^[75]^ EA’s neurorestorative effects of EA are likely mediated by electrophysiological modulation, wherein electrical stimulation upregulates neurotrophic factor expression, accelerates the clearance of degenerative byproducts, and facilitates axonal regeneration and myelin repair.^[76]^ This mechanistic specificity may explain the distinct efficacy of EA in the recovery of motor function.

### 4.3. Relation to previous work

Previous studies have demonstrated that the provision of additional acupuncture interventions dramatically enhances the efficacy of mecobalamine treatment, and some major guidelines recommend the use of MA, HF, or EA for the treatment of DPN. There is still a lack of evidence on the efficacy of acupuncture interventions in patients with DPN receiving mecobalamine. Our study focused on DPN patients receiving mecobalamine, and the results revealed that AI, HF, MA, MA-HF, MA-MOX, MA-TCM, and MOX, as well as AI-TCM and EA, were more effective than mecobalamine was and provide new evidence on the efficacy of multiple acupuncture interventions for DPN patients receiving mecobalamine. This study systematically quantified the relative efficacy of diverse acupuncture modalities in patients with DPN. Our findings highlight the potential of combination therapies, aligned with Yang et al’s theoretical framework on multimodal synergy.^[77]^

### 4.4. Implications for clinical practice and future research

These findings may provide guidance for selecting optimal acupuncture regimens in DPN management. EA is prioritized for rapid motor function restoration, whereas AI-TCM is recommended for sensory function restoration. Although AI-TCM and EA have demonstrated superior efficacy in improving certain functions, other interventions (such as mecobalamine, high-frequency stimulation, and acupoint massage) have also shown certain therapeutic effects. Therefore, clinicians can develop individualized comprehensive treatment plans by combining multiple interventions based on the specific conditions of the patients, thereby enhancing therapeutic outcomes. Future research should also focus on improving the study quality, including the use of rigorous randomization methods, blinding designs, and more precise outcome measures. Moreover, studies involving diverse populations and regions should be conducted to increase the generalizability of the findings. While this study focused primarily on comparing the therapeutic effects of various interventions, future research could further explore the underlying mechanisms by which AI-TCM and EA improve common peroneal nerve function. For example, neurophysiological and molecular biological approaches can be used to elucidate their impact on nerve repair and regeneration.

### 4.5. Strengths and limitations of this study

Our network meta-analysis takes advantage of all direct and indirect comparisons simultaneously, thus making the estimates more precise and consistent. This study has several advantages. First, the study method was concerned with precision and our researchers received strict training. Second, given that most RCTs of acupuncture interventions for DPN are not blinded, which increases the risk of performance bias, the utilization of objective electrophysiological outcomes may mitigate the detection bias inherent in the unblinded design. To our knowledge, this network meta-analysis is the first to compare multiple acupuncture interventions in patients with DPN. Different types of acupuncture intervention are associated with different outcomes in patients with DPN. Our study revealed that, among acupuncture interventions, AI-TCM and EA might be the most effective treatments for improving common peroneal nerve function in patients with DPN. This study had several limitations that should be considered when interpreting the results. First, there are few direct comparisons between the interventions. Second, the control group in some RCTs not only received mecobalamine as usual treatment but also included other medicines, which may have contributed to the statistical heterogeneity and certainty of clinical heterogeneity. Third, although we can optimize the use of all available data through network meta-analyses, indirect evidence is not directly based on RCTs.

## 5. Conclusion

This network meta-analysis identified AI-TCM and EA as optimal adjunctive interventions for patients with DPN receiving conventional therapy. Both modalities demonstrated statistically superior efficacy over mecobalamin monotherapy in improving the motor and sensory nerve function of the compromised common peroneal nerve. Notably, these acupuncture protocols might address a critical therapeutic gap given the paucity of standardized rehabilitation strategies targeting functional recovery in patients with common peroneal nerve injury. While these findings highlight the synergistic therapeutic potential, further trials with extended follow-up periods and mechanistic investigations are needed to establish evidence in clinical practice.

## Author contributions

**Conceptualization:** Shuxiong Lin, Yu Qin, Liming Lu.

**Data curation:** Miaomiao Li, Shuxiong Lin, Maohuai Zhu, Yifen Liu, Huijing Lin.

**Formal analysis:** Miaomiao Li, Yu Qin, Maohuai Zhu, Hao Wen, Yifen Liu.

**Funding acquisition:** Shuxiong Lin, Yu Qin, Liming Lu.

**Investigation:** Yifen Liu, Huijing Lin.

**Methodology:** Yu Qin, Liming Lu.

**Resources:** Shuxiong Lin.

**Software:** Miaomiao Li, Hao Wen.

**Supervision:** Liming Lu.

**Writing – original draft:** Miaomiao Li, Shuxiong Lin, Hao Wen.

## Supplementary Material


